# PERMIT study: a global pooled analysis study of the effectiveness and tolerability of perampanel in routine clinical practice

**DOI:** 10.1007/s00415-021-10751-y

**Published:** 2021-08-24

**Authors:** Vicente Villanueva, Wendyl D’Souza, Hiroko Goji, Dong Wook Kim, Claudio Liguori, Rob McMurray, Imad Najm, Estevo Santamarina, Bernhard J. Steinhoff, Pavel Vlasov, Tony Wu, Eugen Trinka, the PERMIT pooled analysis participants

**Affiliations:** 1 Refractory Epilepsy Unit, Hospital Universitario y Politécnico La Fe, Valencia, Spain; 2 Department of Medicine, St Vincent’s Hospital Melbourne, The University of Melbourne, Victoria, Australia; 3 Neuropsychiatric Department, Aichi Medical University, Aichi, Japan; 4 Department of Neurology, Konkuk University School of Medicine, Seoul, Korea; 5 Epilepsy Centre, Neurology Unit, University Hospital “Tor Vergata”, Rome, Italy; 6 Department of Systems Medicine, University of Rome “Tor Vergata”, Rome, Italy; 7 European Knowledge Centre, Eisai Europe Ltd, Hatfield, Hertfordshire UK; 8 Cleveland Clinic Epilepsy Center, Neurological Institute, Cleveland Clinic, Cleveland, OH USA; 9 Epilepsy Unit, Hospital Universitari Vall D’Hebron, Barcelona, Spain; 10 Kork Epilepsy Center, Kehl-Kork, Germany; 11 Department of Neurology and Neurophysiology, Albert-Ludwigs University of Freiburg, Freiburg, Germany; 12 Department of Neurology, General Medical Faculty of Moscow State University of Dentistry named after A.I. Evdokimov, Moscow, Russian Federation; 13 Chang Gung Memorial Hospital Linkou Medical Center and Chang Gung University College of Medicine, Taoyuan, Taiwan; 14 Department of Neurology, Christian-Doppler University Hospital, Paracelsus Medical University, Affiliated EpiCARE Partner, Centre for Cognitive Neuroscience, Salzburg, Austria; 15 Department of Public Health, Health Services Research and Health Technology Assessment, UMIT-University for Health Sciences, Medical Informatics and Technology, Hall in Tirol, Austria; 16 Neuroscience Institute, Christian Doppler University Hospital, Paracelsus Medical University, Salzburg, Austria

**Keywords:** Antiseizure medication, Effectiveness, Focal epilepsy, Generalized epilepsy, Observational study, Tolerability

## Abstract

The PERaMpanel pooled analysIs of effecTiveness and tolerability (PERMIT) study was a pooled analysis of data from 44 real-world studies from 17 countries, in which people with epilepsy (PWE; focal and generalized) were treated with perampanel (PER). Retention and effectiveness were assessed after 3, 6, and 12 months, and at the last visit (last observation carried forward). Effectiveness assessments included 50% responder rate (≥ 50% reduction in seizure frequency from baseline) and seizure freedom rate (no seizures since at least the prior visit); in PWE with status epilepticus, response was defined as seizures under control. Safety and tolerability were assessed by evaluating adverse events (AEs) and discontinuation due to AEs. The Full Analysis Set included 5193 PWE. Retention, effectiveness and safety/tolerability were assessed in 4721, 4392 and 4617, respectively. Retention on PER treatment at 3, 6, and 12 months was 90.5%, 79.8%, and 64.2%, respectively. Mean retention time on PER treatment was 10.8 months. The 50% responder rate was 58.3% at 12 months and 50.0% at the last visit, and the corresponding seizure freedom rates were 23.2% and 20.5%, respectively; 52.7% of PWE with status epilepticus responded to PER treatment. Overall, 49.9% of PWE reported AEs and the most frequently reported AEs (≥ 5% of PWE) were dizziness/vertigo (15.2%), somnolence (10.6%), irritability (8.4%), and behavioral disorders (5.4%). At 12 months, 17.6% of PWEs had discontinued due to AEs. PERMIT demonstrated that PER is effective and generally well tolerated when used to treat people with focal and/or generalized epilepsy in everyday clinical practice.

**Supplementary Information:**

The online version contains supplementary material available at 10.1007/s00415-021-10751-y.

## Introduction

Perampanel (PER) is the first-in-class, highly selective, non-competitive α-amino-3-hydroxy-5-methyl-4-isoxazolepropionic acid (AMPA) receptor antagonist that inhibits the postsynaptic binding of glutamate by selectively targeting AMPA receptors, which are mainly located on postsynaptic neurons [1–4]. PER is widely indicated for both focal-onset seizures and generalized-onset tonic–clonic seizures in patients with idiopathic generalized epilepsy (IGE). Its approved use in different age groups, and as monotherapy or adjunctive therapy, varies between countries and regions [5–7].

Approval for the treatment of focal-onset seizures was based primarily on the results of three Phase III randomized, double-blind, placebo-controlled trials [8–10] and one pediatric open-label Phase III trial [11]. Approval for the treatment of generalized-onset tonic–clonic seizures in IGE was based on the findings of one Phase III, randomized, double-blind, placebo-controlled trial [12]. Real-world clinical practice data complement evidence from clinical trials by providing information on people with epilepsy (PWE) who are more diverse in terms of clinical characteristics than those recruited for clinical trials [13–15]. In addition, they provide pragmatic information on the dosing and titration schedules employed in clinical practice, which are individualized and applied on a patient-by-patient basis, rather than according to a pre-defined clinical trial protocol [14]. The objective of this study was to investigate the effectiveness, safety and tolerability of PER when used in everyday clinical practice.

## Methods

### Study design

The **PER**a**M**panel pooled analys**I**s of effec**T**iveness and tolerability (PERMIT) study was a pooled analysis of individual patient data from real-world prospective, retrospective and cross sectional studies and work groups in which people with focal and generalized epilepsy were treated with PER. The studies were identified by a systematic PubMed literature search, supported by searches of abstracts from key epilepsy congresses from 2012 to December 2019. There were no exclusion criteria for the studies or work groups included in the analysis in terms of the epilepsy type of those studied and/or the number of prior antiseizure medications (ASMs) they had received. The only exclusion criterion for the overall study was that the principal investigator of the respective study did not agree to participate in the pooled analysis. De-identified data from individual PWE were pooled together for baseline number of seizures, type of epilepsy/seizures, prior ASMs, dosage, effectiveness at various time points, and adverse events (AEs). Effectiveness was assessed after 3, 6, and 12 months of PER treatment and at final follow-up (i.e. the last observation of each PWE, independent of the timepoint when it occurred [last observation carried forward]; defined as ‘last visit’). Safety and tolerability were assessed for the duration of PER treatment.

Each study included in PERMIT was approved by its own independent ethics committee, which was subsequently informed, if needed by local legislation, about the PERMIT pooled analysis.

### Study population

PERMIT included PWE from studies and work groups conducted in 17 countries in Europe, Asia, North America, the Middle East and Australasia (Table 1). Details of the specific inclusion/exclusion criteria used in the individual studies have been published or presented previously [16–56]. Studies included in the analysis employed broad inclusion/exclusion criteria, to be representative of PWE encountered in clinical practice. All PWE from these studies who initiated PER for the treatment of epilepsy were included in the pooled analysis. PWE were excluded if records contained insufficient data for analysis. PWE from publications by the same authors and the same geographic areas were compared, and PWE with the same baseline characteristics, treatment start/end date, information at follow-up visits, and treatment completion were excluded, in order to ensure that there were no duplicate data.

**Table 1:** Overview of studies included in the pooled analysis

Study name (if applicable) Reference	Country/countries	Design	Population	Number of PWE
*Published articles*				
Rohracher et al. [16]	Austria, France, Germany, Italy, Spain, Sweden, UK	Multicenter Pooled observational data	Several populations typically under-represented in clinical trials	2396
Kim and Oh [17]	Korea	Single-center Prospective	PWE aged ≥ 17 years	137
PERADON Abril Jaramillo et al. [18]	Spain	Multicenter Prospective	PWE aged ≥ 12 years with FOS and PER as first add-on therapy	113
Santamarina et al. [19]	Spain	Multicenter Retrospective	PWE aged ≥ 12 years and PER as first add-on therapy	144
GENERAL Villanueva et al. [20]	Spain	Multicenter Retrospective	People aged ≥ 12 years with IGE	149
PERADET Coppola et al. [21]	Italy	Multicenter Prospective	Adult PWE with brain tumor	14
Toledo et al. [22]	Spain	Multicenter Prospective	PWE aged ≥ 16 years with FOS (sleep quality study)	72
Strzelczyk et al. [23]	Austria, Finland, Germany, Spain	Multicenter Retrospective	Adults with status epilepticus	52
Gil-López et al. [24]	Spain	Multicenter Retrospective	PWE aged ≥ 16 years with myoclonic seizures	31
Auvin et al. [25]	France	Single center Prospective	Children and adults with Lennox-Gastaut syndrome	13
Alsaadi et al. [26]	United Arab Emirates	Single center Retrospective	People with IGE treated with PER monotherapy	21
Izumoto et al. [27]	Japan	Single center Prospective	Adult PWE with brain tumor	12
Deleo et al. [28]	Italy	Multicenter Prospective	PWE aged ≥ 16 years with drug-resistant focal epilepsy	56
Maschio et al. [29]	Italy	Multicenter Retrospective	Adult PWE with brain tumor	27
Steinhoff et al. [30]	Germany	Single center Retrospective	Adult PWE	92
Rea et al. [31]	Italy	Multicenter Retrospective	Adult PWE and PER as first add-on therapy	27
Toledano et al. [32]	Spain	Multicenter Retrospective	PWE aged ≥ 12 years and PER monotherapy	98
Moraes et al. [33]	Australia	Prospective Retrospective	Adult PWE	93
Chiang et al. [34]	Taiwan	Multicenter Retrospective	PWE aged ≥ 16 years	229
Datta et al. [35]	Canada	Single center Retrospective	Pediatric population with epilepsy	24
Coyle et al. [36]	UK	Single center Retrospective	Adult PWE	23
Ho et al. [37]	Taiwan	Single center Retrospective	PWE with refractory status epilepticus	22
De Liso et al. [38]	Italy	Multicenter Retrospective	Children and adolescents with refractory epilepsy	62
Liguori et al. [39, 40]	Italy	Single center Retrospective	Adult PWE	96
Takahashi et al. [41]	Japan	Multicenter Retrospective	PWE with FOS and PER as early add-on vs. late add-on therapy	51
Morano et al. [42]	Italy	Single center Retrospective	PWE with highly refractory epilepsy	89
Goji and Kanemoto [43]	Japan	Single center Prospective	Adult PWE	128
Rocamora et al. [44]	Spain	Retrospective	Adult PWE	77
Gil-Nagel et al. [45]	Spain	Multicenter Retrospective	Monotherapy	60
Vlasov et al. [46]	Russia	Multicenter Retrospective	PWE with focal epilepsy	164
*Abstracts*				
Teijeira et al. [47]	Spain	Single center	PWE aged ≥ 12 years and PER as first add-on therapy or monotherapy	26
Ron et al. [48]	Spain	Multicenter Prospective	Children with epilepsy	14
Carreño et al. [49]	Spain	Single center Retrospective	PWE with seizures exclusively during sleep	98
Yamamoto [50]	Japan	Retrospective	PWE with PER as early add-on vs. late add-on therapy	70
Pereagal Osorio et al. [51]	Spain	Multicenter Retrospective	Adult PWE with FOS and PER as early add-on therapy	77
Odintsova [52]	Russia	–	PWE	49
Matricardi et al. [53]	Italy	Multicenter Retrospective	PWE with FOS and Lennox-Gastaut syndrome	58
Valente Fernandes (unpublished^a^)	Portugal	–	Brain tumor	7
Chinvarun [54]	Thailand	–	PWE and PER monotherapy	41
Kristensen [55]	Norway	Single center Retrospective	PWE with FOS and PGTCS	44
Bonanni et al. [56]	Italy	–	PWE with intellectual disability	55
*Local databases*				
Morillo^b^	Canada	–	Adult PWE	41
Suller Marti^c^	Canada	–	Adult PWE	5
Jacobs-LeVan^d^	Canada	–	–	36

*FOS* focal-onset seizures, *IGE* idiopathic generalized epilepsy, *PER* perampanel, *PGTCS* primary generalized tonic–clonic seizures, *PWE* people with epilepsy

^a^Presented at Liga Portuguesa Contra a Epilepsia (LPCE) congress 2019

^b^Hamilton General Hospital, Hamilton, Ontario, Canada (local database)

^c^London Health Science Center, London, Ontario, Canada (local database)

^d^Alberta Children’s Hospital, Calgary, Alberta, Canada (local database)

### Study assessments

Retention was assessed after 3, 6 and 12 months of PER treatment. Long-term retention (defined as > 12 months) was also assessed for those studies that reported it. Effectiveness was assessed after 3, 6 and 12 months and at the last visit. In individuals with focal or generalized seizures, effectiveness assessments included change from baseline in seizure frequency (by seizure type), 50% responder rate (response defined as ≥ 50% reduction in seizure frequency from baseline), seizure freedom rate (seizure freedom defined as no seizures since at least the prior visit), and the proportion of PWE with worsened seizure frequency. At baseline (i.e., prior to PER initiation), monthly seizure frequency was defined based on the criteria used for each individual study. At other timepoints, monthly seizure frequency was based on the number of seizures experienced since the previous visit. For the final assessment, monthly seizure frequency was based on the last visit, which could have been at 3, 6, or 12 months; therefore, seizure frequency at the last visit was based on the number of seizures experienced during at least the previous 3 months. In individuals with status epilepticus, effectiveness was assessed as responder rate, where response was defined as seizures under control; this meant that the patient responded to PER treatment and their seizures remained under control with its use (although the duration of control was not reported). Safety and tolerability were assessed by evaluating the number and type of AEs, AEs leading to discontinuation, psychiatric AEs, and psychiatric AEs leading to discontinuation. Additional assessments included evaluation of information relating to PER dosing and titration, and changes in the number of concomitant ASMs between baseline and the last visit.

### Statistical analyses

#### Analysis populations

The Full Analysis Set (FAS) comprised all PWE who were treated with PER. The Retention Population included PWE from the FAS population whose PER status was known at some point during the first 12 months after starting treatment (including those with ongoing PER treatment at 12 months, those who stopped PER prior to 12 months, and those lost to follow-up or end of study follow-up prior to 12 months). The Effectiveness Population included PWE from the FAS population who had at least one effectiveness measurement available. The Tolerability Population included PWE from the FAS population for whom data on AEs were available.

#### Statistical considerations and methods

There was great heterogeneity in the particular objectives of each study included in the pooled analysis and therefore in the information that each study reported. The current study attempted to combine the reported information in the most complete and harmonized way possible. If patients had effectiveness assessed, their measurements were collected until they discontinued or were lost to follow-up. Missing data were not imputed, except in cross-sectional studies, in which the last visit datum was captured to include it in the established cut-off points (3, 6 or 12 months). When the observation timepoint of the study did not match the established cut-off points, the following allocations were made: observations performed between 1.5–4.5 months were allocated to the 3-month visit; those performed between 4.5–9 months were allocated to the 6-month visit; and those performed between 9–15 months were allocated to the 12-month visit. A ‘final’ variable was created in which the last observation of each PWE was included (last observation carried forward), independently of the moment when it occurred (defined as ‘last visit’). Since the studies included in PERMIT did not have a common objective and varied in terms of patient selection criteria, no hypothesis was defined, meta-analysis of individualized PWE data was not conducted, and the individual studies were not treated as clusters.

A descriptive analysis of recorded quantitative and qualitative variables was performed. Quantitative variables were described as mean, standard deviation (SD), median, minimum and maximum values, together with the number of valid cases and confidence intervals (CIs) or interquartile range (25th percentile to 75th percentile). Qualitative variables were described as absolute frequencies and percentages. Data were not available for all PWE at every time point; therefore, for each variable, the total number of PWE for whom the data in question were available was stated and this value was used as the denominator for frequency analyses. Retention (on PER treatment) was studied within the first 12 months of follow-up using Kaplan–Meier methodology. Variation in the number of seizures per month between baseline and the last visit was assessed using the Wilcoxon signed-rank test and variation in the type of seizures was assessed using McNemar’s test. The signification level was established at 5%. Statistical package SPSS 25.0 was used for all analyses.

#### Bivariate and multivariate analyses

Binary logistic regression analyses were performed to study possible factors associated with retention, effectiveness (seizure freedom, response) and tolerability. Potential relationships between baseline characteristics (subject- and therapy-related factors) and the dependent variables (retention, seizure freedom, response, tolerability) were determined by bivariable methods, using the Chi-squared test, Student’s *t*-test or Mann–Whitney *U* test, as appropriate, to determine statistical significance. Baseline characteristics included sex, age, age at epilepsy onset, duration of epilepsy, etiology, presence of learning disability, presence of psychiatric comorbidity, seizure type, use of PER as early or late add-on therapy, use of PER monotherapy, rate of PER titration (fast [2 mg/week]/slow [less than 2 mg/week]), PER dosage (≤ 4 mg/day, ≤ 6 mg/day), number of previous ASMs, and number and types of concomitant ASMs. Baseline characteristics with a p-value of < 0.10 were pre-selected to construct exploratory multivariable binary logistic regression models for retention, effectiveness and tolerability.

## Results

Information was gathered from 5200 PWE who had initiated treatment with PER. Two PWE were excluded because they never started treatment and five PWE because they were included in two studies. The final FAS therefore included 5193 individual PWE from a total of 44 real-world studies/work groups from a wide range of countries, details of which are presented in Table 1 [16–56].

**Fig. 1:**
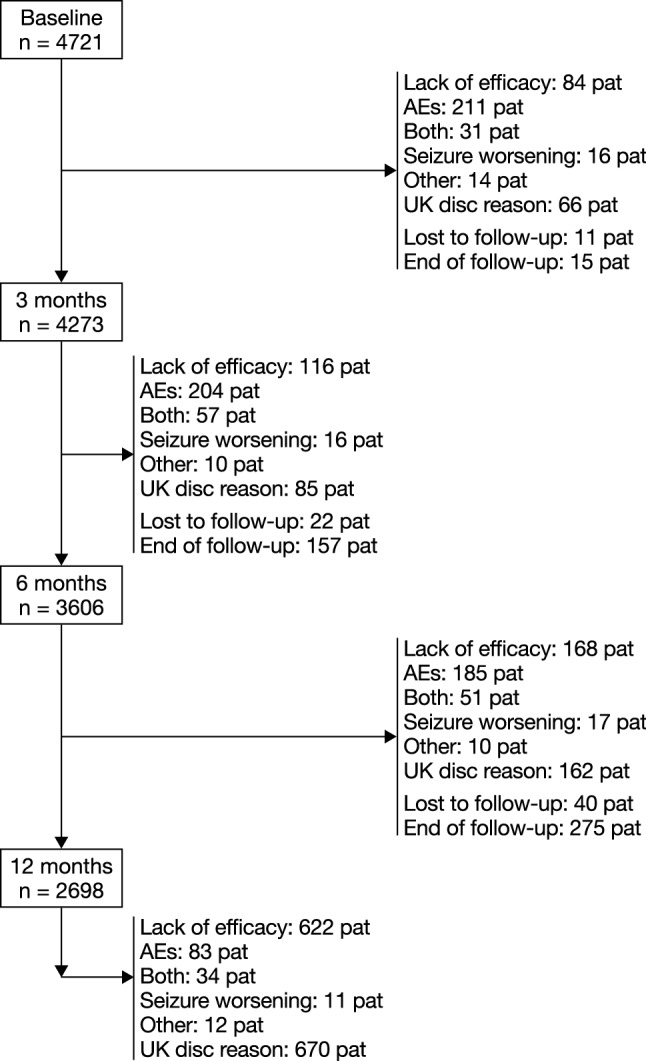
Disposition of PWE (Retention Population). *AE* adverse event, *pat* patients, *PWE* people with epilepsy, *UK* unknown

Disposition was assessed for the Retention Population, which included 4721 PWE (Fig. 1). Overall, 2698 PWE (57.1%) completed at least 12 months of PER treatment. The Effectiveness Population included 4392 PWE and the Tolerability Population included 4617 PWE.

### Study population

Demographic and baseline characteristics are presented in Table 2. In the FAS, 50.5% of PWE were female, the mean (SD) age was 39.7 (16.2) years and the majority of PWE (85.6%) were aged ≥ 18 to < 65 years. Mean (SD) duration of epilepsy was 23.5 (16.0) years and 52.7% of PWE had a structural etiology (International League Against Epilepsy 2017 classification [57]). The median number of previous ASMs PWE had received was 4.0 (range 0‒19; mean 4.9; SD, 3.9) and the median number of concomitant ASMs at baseline was 2.0 (range 0‒7; mean 2.2; SD, 1.2). Demographic and baseline characteristics of PWE in the retention, effectiveness and tolerability populations were generally similar to those of the FAS population (Table 2).

**Table 2:** Demographic and baseline characteristics in the Full Analysis Set, Retention Population, Effectiveness Population and Tolerability Population

Characteristic	Full Analysis Set	Retention Population	Effectiveness Population	Tolerability Population
	*N* = 5193	*N* = 4721	*N* = 4392	*N* = 4617
Sex				
* N*^a^	5175	4717	4376	4602
Female, *n* (%)	2612 (50.5)	2365 (50.1)	2182 (49.9)	2348 (51.0)
Male, *n* (%)	2563 (49.5)	2352 (49.9)	2194 (50.1)	2254 (49.0)
Age				
* N*^a^	4946	4555	4309	4443
Mean (SD), years	39.7 (16.2)	39.6 (15.9)	39.5 (16.2)	39.7 (16.1)
Median (range), years	38.6 (2.0‒97.0)	38.0 (2.0‒97.0)	38.0 (2.0‒92.0)	38.0 (2.0‒97.0)
Age category				
*N*^a^	5006	4606	4309	4503
< 12 years, *n* (%)	64 (1.3)	48 (1.0)	50 (1.2)	55 (1.2)
≥ 12‒ < 18 years, *n* (%)	279 (5.6)	266 (5.8)	258 (6.0)	258 (5.7)
≥ 18‒ < 65 years, *n* (%)	4284 (85.6)	3964 (86.1)	3681 (85.4)	3854 (85.6)
≥ 65 years	379 (7.6)	328 (7.1)	320 (7.4)	336 (7.5)
Age at epilepsy onset				
*N*^a^	4122	‒	‒	‒
Mean (SD), years	16.1 (17.2)			
Median (range), years	12.0 (0.0‒97.0)			
Duration of epilepsy				
*N*^a^	4228	4051	3632	3748
Mean (SD), years	23.5 (16.0)	23.5 (16.0)	23.4 (16.0)	23.7 (16.1)
Median (range), years	21.0 (0.0‒82.0)	21.0 (0.0‒82.0)	21.0 (0.0‒82.0)	21.9 (0.0‒82.0)
Etiology^b^				
*N*^a^	3830	‒	‒	‒
Structural, *n* (%)	2013 (52.6)			
Unknown, *n* (%)	1199 (31.3)			
Genetic, *n* (%)	475 (12.4)			
Infectious, *n* (%)	107 (2.8)			
Immune, *n* (%)	31 (0.8)			
Metabolic, *n* (%)	3 (0.1)			
Other, *n* (%)	2 (0.1)			
Epilepsy syndrome				
*N*^a^	2495	‒	‒	‒
No, *n* (%)	2037 (81.6)			
Yes, *n* (%)	458 (18.4)			
Vascular etiology				
*N*^a^	2495	‒	‒	‒
No, *n* (%)	2371 (95.0)			
Yes, *n* (%)	124 (5.0)			
Tumor etiology				
*N*^a^	2495	‒	‒	‒
No, *n* (%)	2360 (94.6)			
Yes, *n* (%)	135 (5.4)			
Learning disability				
*N*^a^	2626	‒	‒	‒
No, *n* (%)	1890 (72.0)			
Yes, *n* (%)	736 (28.0)			
Psychiatric comorbidity				
*N*^a^	2661	‒	‒	‒
No, *n* (%)	2027 (76.2)			
Yes, *n* (%)	634 (23.8)			
Most frequent types^c^ of psychiatric comorbidity				
*N*^a^	2661	‒	‒	‒
Depression, *n* (%)	111 (4.2)			
Anxiety, *n* (%)	89 (3.3)			
Hyperactivity, *n* (%)	26 (1.0)			
Seizure type				
*N*^a^	4083	‒	‒	‒
Focal	3911 (81.4)			
Generalized	604 (12.6)			
Both focal and generalized	214 (4.5)			
Status epilepticus	74 (1.5)			
Number of previous ASMs				
*N*^a^	3999	3760	3407	3568
Mean (SD)	4.9 (3.9)	4.9 (3.9)	4.9 (3.9)	4.8 (3.9)
Median (range)	4.0 (0‒19)	4.0 (0‒19)	4.0 (0‒19)	4.0 (0‒19)
Number of previous ASMs				
*N*^a^	3999	‒	‒	‒
0, *n* (%)	451 (11.3)			
1, *n* (%)	428 (10.7)			
2, *n* (%)	459 (11.5)			
3, *n* (%)	405 (10.1)			
4, *n* (%)	353 (8.8)			
5, *n* (%)	296 (7.4)			
≥ 6, *n* (%)	1607 (40.2)			
Most frequently used^d^ previous ASMs				
*N*^a^	1332	‒	‒	‒
Levetiracetam, *n* (%)	330 (24.8)			
Valproate, *n* (%)	300 (22.5)			
Carbamazepine, *n* (%)	261 (19.6)			
Topiramate, *n* (%)	261 (19.6)			
Lamotrigine, *n* (%)	182 (13.7)			
Phenytoin, *n* (%)	170 (12.8)			
Number of concomitant ASMs				
*N*^a^	4916	4561	4185	4344
Mean (SD)	2.2 (1.2)	2.3 (1.1)	2.2 (1.1)	2.3 (1.1)
Median (range)	2.0 (0‒7)	2.0 (0‒7)	2.0 (0‒7)	2.0 (0‒7)
Number of concomitant ASMs				
*N*^a^	4916	‒	‒	‒
0, *n* (%)	269 (5.5)			
1, *n* (%)	1031 (21.0)			
2, *n* (%)	1681 (34.2)			
3, *n* (%)	1313 (26.7)			
≥ 4, *n* (%)	622 (12.7)			
Most frequently used^d^ concomitant ASMs				
*N*^a^	4756	‒	‒	‒
Levetiracetam, *n* (%)	1777 (37.4)			
Valproate, *n* (%)	1172 (24.6)			
Lamotrigine, *n* (%)	1120 (23.5)			
Carbamazepine, *n* (%)	1028 (21.6)			
Lacosamide, *n* (%)	930 (19.6)			
Clobazam, *n* (%)	750 (15.8)			
Zonisamide, *n* (%)	617 (13.0)			
Topiramate, *n* (%)	507 (10.7)			
Oxcarbazepine, *n* (%)	486 (10.2)			
Types of concomitant ASMs				
*N*^a^	4756	4408	4028	4184
Enzyme inducers,^e^ *n* (%)	2365 (49.7)	2250 (51.0)	2048 (50.8)	2134 (51.0)
Sodium channel blockers,^f^ *n* (%)	3368 (70.8)	‒	‒	‒
ASMs targeting the GABA system,^g^ *n* (%)	1507 (31.7)	‒	‒	‒
Calcium channel blockers,^h^ *n* (%)	254 (5.3)	‒	‒	‒
Potassium channel blockers,^i^ *n* (%)	93 (2.0)	‒	‒	‒
Synaptic vesicle protein-2 modulators,^j^ *n* (%)	1809 (38.0)	‒	‒	‒
ASMs with mixed mode of action,^k^ *n* (%)	2022 (42.5)	‒	‒	‒

*ASM* antiseizure medication, *GABA* gamma aminobutyric acid, *PWE* people with epilepsy, *SD* standard deviation

^a^Number of PWE for whom datum in question was available

^b^International League Against Epilepsy 2017 classification

^c^ ≥ 1% of PWE

^d^ ≥ 10% of PWE

^e^Defined as carbamazepine, eslicarbazepine acetate, oxcarbazepine, phenobarbital, phenytoin and primidone

^f^Defined as carbamazepine, eslicarbazepine acetate, lacosamide, lamotrigine, oxcarbazepine, phenytoin and rufinamide

^g^Defined as clobazam, clonazepam, diazepam, phenobarbital, primidone, tiagabine and vigabatrin

^h^Defined as ethosuximide, gabapentin and pregabalin

^i^Defined as retigabine

^j^Defined as brivaracetam and levetiracetam

^k^Defined as felbamate, stiripentol, topiramate, valproate and zonisamide

### PER treatment and concomitant ASMs (FAS)

The mean (SD) baseline PER dose was 2.4 (1.1) mg/day in the overall population (median 2.0; range, 1–20; *n* = 2190). At the end of the observation period (last visit), the mean (SD) PER dose was 6.3 (2.6) mg/day (median 6.0; range 1–24; *n* = 3411). In individuals without status epilepticus, the mean (SD) PER dose was 2.3 (1.1) mg/day (median 2.0; range 1–20; *n* = 2168) at baseline, and 6.3 (2.6) mg/day (median 6.0; range 1–18; *n* = 3339) at the last visit. In those with status epilepticus, the mean (SD) PER dose was 6.6 (3.8) mg/day (median 6.0; range 2–24; *n* = 72). The rate of PER titration was known for 1880 PWE. A fast titration (2 mg/week) was used in 65.5% of PWE (1231/1880) and a slow titration (less than 2 mg/week) was used in 34.5% of PWE (649/1880). The mean (SD) number of concomitant ASMs was 2.2 (1.2) mg/day (median 2.0; range 0–7; *n* = 4916) at baseline and 2.0 (1.0) mg/day (median 2.0; range 0–6; *n* = 1832) at the last visit. The proportion of PWE treated with PER monotherapy was 5.6% (269/4816) at baseline and 4.1% (76/1832) at the last visit.

### Retention (Retention Population)

**Fig. 2:**
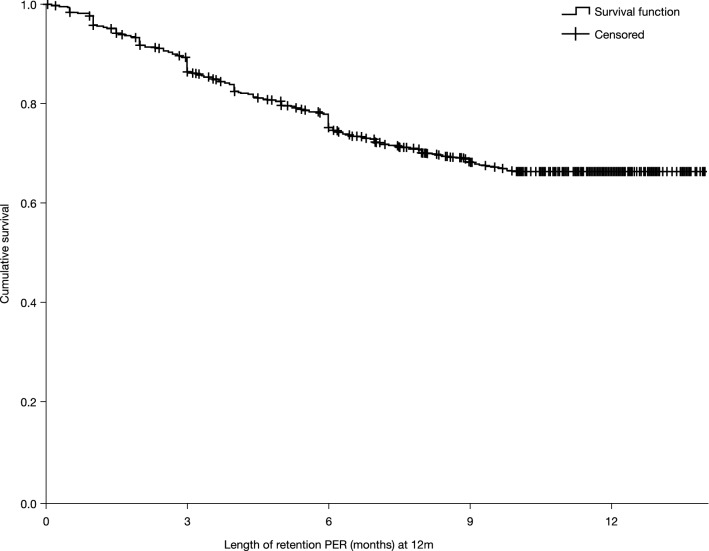
Kaplan–Meier curve for retention on PER treatment over 12 months (Retention Population). *PER* perampanel

Retention on PER treatment at 3, 6, and 12 months was 90.5% (4273/4721), 79.8% (3603/4516), and 64.2% (2698/4201), respectively. The mean retention time on PER treatment was 10.8 months (95% CI 10.6–10.9; Kaplan–Meier analysis; Fig. 2). It was not possible to estimate the median retention time, due to the high number of censored events. Reasons for discontinuation over 12 months were AE(s) (14.3%; 600/4201), lack of efficacy (8.8%; 368/4201), both AE(s) and lack of efficacy (3.3%; 139/4201), seizure worsening (1.2%; 49/4201), other reasons (0.8%; 34/4201; death, *n* = 9; financial problems, *n* = 7; pregnancy, *n* = 4; PWE decision—not otherwise specified, *n* = 4; disease progression [tumor], *n* = 4; transferred to another hospital, *n* = 2; surgery, *n* = 1; unable to take medicine [pneumonia], *n* = 1; poor compliance, *n* = 1; interaction with tuberculosis medication, *n* = 1) and unknown (7.5%; 313/4201). Over the longer term (> 12 months), retention was 29.5% (1229/4164) and the mean retention time on PER treatment was 18.7 months (95% CI, 17.8–19.5). Reasons for discontinuation over the longer term were lack of efficacy (23.8%; 990/4164), AE(s) (16.4%; 683/4164), both AE(s) and lack of efficacy (4.2%; 173/4164), seizure worsening (1.4%; 60/4164), other reasons (1.1%; 46/4164; death, *n* = 10; financial problems, *n* = 8; pregnancy, *n* = 6; improvement, *n* = 5; PWE decision—not otherwise specified, *n* = 4; disease progression [tumor], *n* = 4; surgery, *n* = 2; transferred to another hospital, *n* = 2; accident, *n* = 1; prior myocardial infarction, *n* = 1; unable to take medicine [pneumonia], *n* = 1; poor compliance, *n* = 1; interaction with tuberculosis medication, *n* = 1) and unknown (23.6%; 983/4164).

**Fig. 3:**
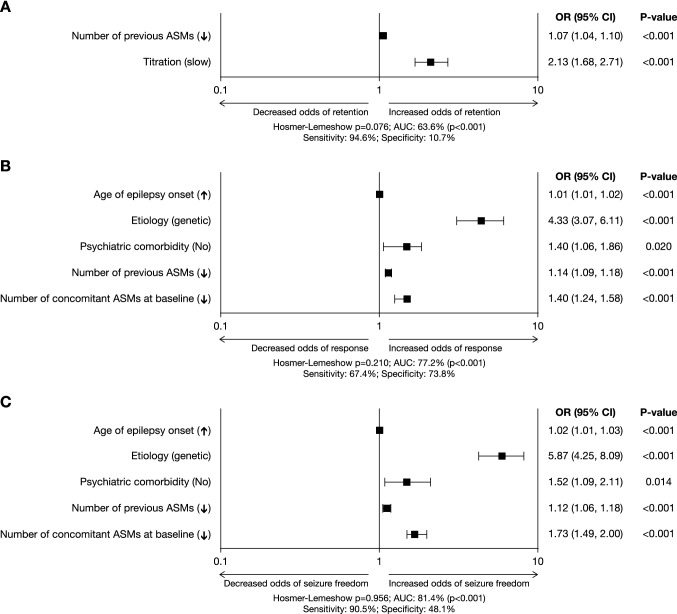
Multivariate analyses of relationships between baseline characteristics and **a** retention (Retention Population), **b** response to PER treatment (Effectiveness Population) and **c** seizure freedom (Effectiveness Population). Seizure freedom was defined as no seizures since at least the prior visit. Response was defined as ≥ 50% seizure frequency reduction from baseline. *ASM* antiseizure medication, *AUC* area under the curve, *CI* confidence interval, *OR* odds ratio

Results of bivariable analysis of the associations of baseline characteristics with retention at 12 months are presented in Supplementary Table 1 (baseline characteristics selected for inclusion in the model to predict retention [i.e. those with *p* < 0.10] are indicated as shaded cells). Multivariate binary regression analysis revealed that the baseline characteristics that were most associated with likelihood of retention were lower number of previous ASMs (odds ratio [OR] 1.07 [95% CI 1.04–1.10]; *p* < 0.001) and use of a slow PER titration schedule (OR 2.13 [95% CI 1.68–2.71]) (Fig. 3A). The resulting model had high sensitivity (94.6%) but very low specificity (10.7%).

### Effectiveness (all PWE except those with status epilepticus; Effectiveness Population)

#### Change in seizure frequency by seizure type

**Fig. 4:**
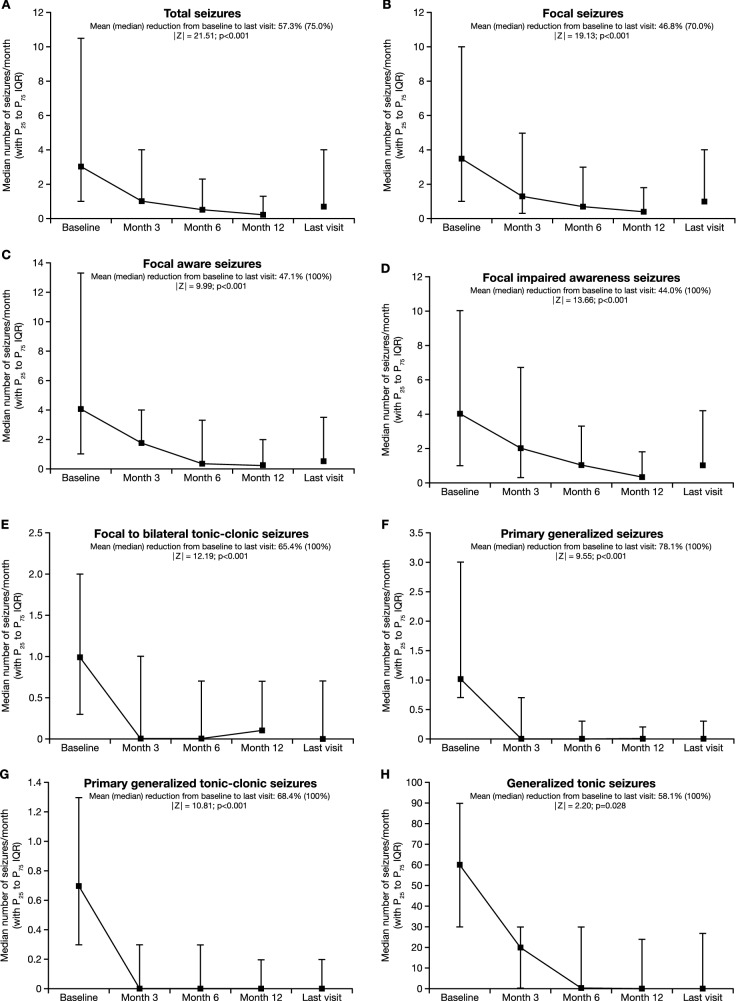

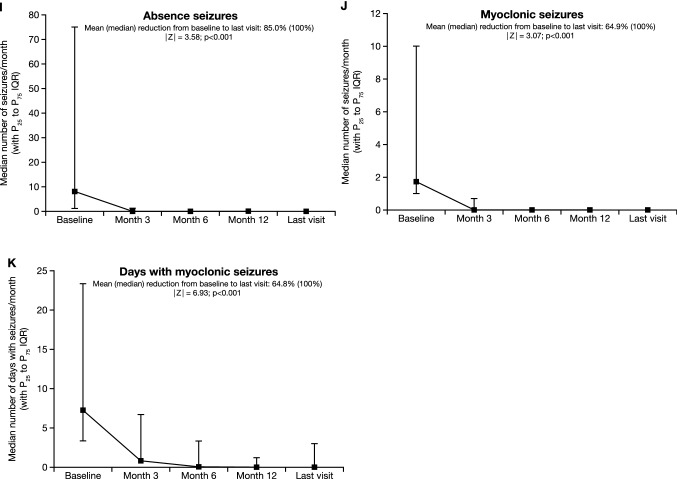
Median monthly frequencies (with P_25_ and P_75_ IQR) at baseline, Month 3, Month 6, Month 12 and the last visit for **a** total seizures, **b** focal seizures, **c** focal aware seizures, **d** focal impaired awareness seizures, **e** focal to bilateral tonic–clonic seizures, **f** primary generalized seizures, **g** primary generalized tonic–clonic seizures, **h** generalized tonic seizures, **i** absence seizures, **j** myoclonic seizures, and **k** days with myoclonic seizures. *IQR* interquartile range, *P* percentile

At the time of PER initiation, 96.3% (1825/1896) of PWE presented having had at least one seizure in the past 3 months. The monthly total seizure frequency decreased significantly from a median of 3.0 (mean [SD], 17.2 [60.6]: range 0.1–1120.0) at baseline to 0.7 (mean [SD], 7.3 [22.2]: range 0.0–300.0) at the last visit (|*Z*|= 21.51; *p* < 0.001; Wilcoxon signed-rank test) (Fig. 4A). The mean reduction from baseline to the last visit was 57.3% (median, 75.0%). The percentage of individuals with focal seizures (i.e., the percentage of PWE who experienced at least one focal seizure during an assessment period of ≥ 3 months) decreased significantly from 71.8% (1167/1626) at baseline to 51.9% (682/1314) at the last visit (*p* < 0.001; McNemar’s test). Similarly, the percentage of PWE with focal aware seizures decreased from 30.2% (313/1037) at baseline to 20.1% (173/861) at the last visit (*p* < 0.001), the percentage of PWE with focal impaired awareness seizures decreased from 55.4% (574/1037) at baseline to 40.3% (347/861) at the last visit (*p* < 0.001), and the percentage of PWE with focal to bilateral tonic–clonic seizures decreased from 41.6% (431/1037) at baseline to 20.8% (179/861) at the last visit (*p* < 0.001). There were significant reductions from baseline to the last visit in the monthly frequencies of focal, focal aware, focal impaired awareness, and focal to bilateral tonic–clonic seizures (mean [median] reductions, 46.8% [70.0%], 47.1% [100%], 44.0% [100%], and 65.4% [100%], respectively; *p* < 0.001 for all [baseline versus last visit]) (Fig. 4B–E).

The percentage of PWE with primary generalized seizures decreased significantly from 24.5% (399/1626) at baseline to 10.8% (142/1314) at the last visit (*p* < 0.001; McNemar’s test). The percentage of PWE with primary generalized tonic–clonic seizures decreased from 76.9% (300/390) at baseline to 25.8% (78/302) at the last visit (*p* < 0.001), the percentage of PWE with generalized tonic seizures decreased from 9.2% (36/390) at baseline to 1.3% (4/302) at the last visit (*p* = not significant), the percentage of PWE with absence seizures decreased from 20.0% (78/390) at baseline to 11.9% (36/302) at the last visit (*p* < 0.001), and the percentage of PWE with myoclonic seizures decreased from 30.3% (118/390) at baseline to 11.9% (36/302) at the last visit (*p* < 0.001). There were significant reductions from baseline to the last visit in the monthly frequencies of generalized, generalized tonic–clonic, generalized tonic, absence and myoclonic seizures, and in the frequency of days with myoclonic seizures (mean [median] reductions, 78.1% [100%], 68.4% [100%], 58.1% [100%], 85.0% [100%], 64.9% [100%], and 64.8% [100%], respectively; *p* < 0.001 for all except generalized tonic seizures [*p* = 0.028] [baseline vs. last visit]) (Fig. 4F–K).

#### Responder rate

**Fig. 5:**
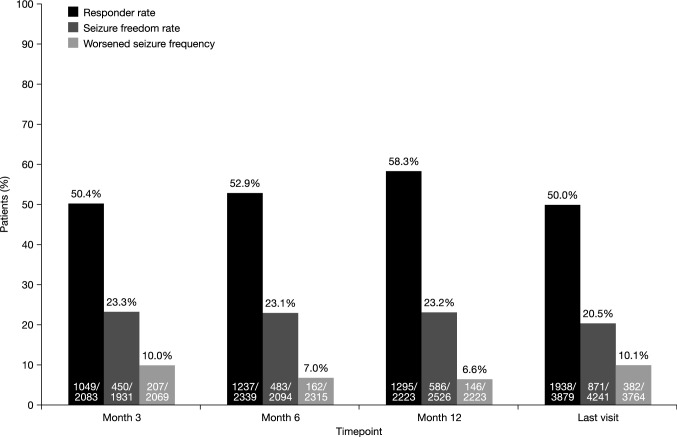
Responder rate, seizure freedom rate, and percentage of PWE with worsened seizure frequency (relative to baseline) at Month 3, Month 6, Month 12 and the last visit. Response was defined as ≥ 50% reduction in seizure frequency from baseline. Seizure freedom was defined as no seizures since at least the prior visit; therefore, seizure freedom rates at Month 3, Month 6 and the last visit represent the percentages of PWE who had no seizures for ≥ 3 months, and the seizure freedom rate at Month 12 represents the percentage of PWE who had no seizures for ≥ 6 months. *PWE* people with epilepsy

The responder rate was 58.3% at 12 months and 50.0% at the last visit (Fig. 5).

Results of bivariable analysis of the associations of baseline characteristics with response during the first 12 months are presented in Supplementary Table 2A (baseline characteristics selected for inclusion in the model to predict response [i.e. those with *p* < 0.10] are indicated as shaded cells). Multivariate binary regression analysis revealed that the baseline characteristics that were most associated with likelihood of response were higher age of onset of epilepsy (OR 1.01 [95% CI 1.01–1.02]; *p* < 0.001), presence of a genetic etiology (OR 4.33 [95% CI 3.07–6.11]; *p* < 0.001), absence of psychiatric comorbidity (OR 1.40 [95% CI 1.06–1.86]; *p* = 0.020), lower number of previous ASMs (OR 1.14 [95% CI 1.09–1.18]; *p* < 0.001), and lower number of concomitant ASMs at baseline (OR 1.40 [95% CI 1.24–1.58]; *p* < 0.001) (Fig. 3B). However, the resulting model had low sensitivity (67.4%) and specificity (73.8%).

#### Seizure freedom rate

The seizure freedom rate was 23.2% at 12 months and 20.5% at the last visit (Fig. 5). A total of 195 PWE presented with no seizures at every recorded timepoint during follow-up (although in some cases only the last visit was recorded). The duration of observation for these PWE ranged from 2.9 to 14 months. Of these 195 PWE, 157 (80.5%) presented with seizures at baseline and 38 (19.5%) did not present with seizures at baseline.

Results of bivariable analysis of the associations of baseline characteristics with seizure freedom during the first 12 months are presented in Supplementary Table 2B (baseline characteristics selected for inclusion in the model to predict seizure freedom [i.e. those with *p* < 0.10] are indicated as shaded cells). Multivariate binary regression analysis revealed that the baseline characteristics that were most associated with likelihood of seizure freedom were higher age at onset of epilepsy (OR 1.02 [95% CI 1.01–1.03]; *p* < 0.001), presence of a genetic etiology (OR 5.87 [95% CI 4.25–8.09]; *p* < 0.001), absence of psychiatric comorbidity (OR 1.52 [95% CI 1.09–2.11]; *p* = 0.014), lower number of previous ASMs (OR 1.12 [95% CI 1.06–1.18]; *p* < 0.001), and lower number of concomitant ASMs at baseline (OR 1.73 [95% CI 1.49–2.00]; *p* < 0.001) (Fig. 3C). The resulting model had high sensitivity (90.5%) but low specificity (48.1%).

#### Percentage of PWE with worsened seizure frequency

The percentages of PWE with worsened seizure frequency, relative to baseline, remained generally stable at all timepoints (Fig. 5). The percentage of PWE with worsened seizure frequency was 6.6% at 12 months and 10.1% at the last visit. The percentage of PWE with focal seizures who had worsening seizure frequency was 7.6% at 12 months and 11.2% at the last visit; in those with generalized seizures, the corresponding values were 2.7% and 5.7%, respectively.

### Effectiveness in PWE with status epilepticus (Effectiveness Population)

A total of 74 PWE in the Effectiveness Population had status epilepticus. Of these PWE, 39 (52.7%) responded to PER treatment (i.e., PER treatment brought status epilepticus under control).

### Safety and tolerability (Tolerability Population)

Overall, 49.9% (2303/4617) of PWE reported AEs at some point during follow-up (Tolerability Population; Table 3). The most frequently reported AEs (≥ 3% of PWE) were dizziness/vertigo (15.2%), somnolence (10.6%), irritability (8.4%), behavioral disorders (5.4%), instability/ataxia (4.1%), and fatigue (3.7%). The incidence of AEs was significantly higher in PWE for whom a fast PER titration was used, compared with those for whom a slow titration was used (61.9% [646/1043] vs. 46.4% [295/636]; χ^2^ = 9.36; *p* = 0.002; Chi-square test). Psychiatric AEs were experienced by 21.0% (965/4590) of PWE. There was a significant association between the incidence of psychiatric AEs and the presence of previous psychiatric comorbidity (χ^2^ = 52.43; *p* < 0.001; Chi-squared test). Of the 965 PWE with psychiatric AEs, the presence of psychiatric comorbidity was known for 509 PWE, of whom 185 (36.3%) had psychiatric comorbidity and 324 (63.7%) did not. Of the 3625 PWE without psychiatric AEs, the presence of psychiatric comorbidity was known for 1809 PWE, of whom 376 (20.8%) had psychiatric comorbidity and 1433 (79.2%) did not. At 12 months, 17.6% (739/4201) of PWEs had discontinued PER due to AEs. Over the longer term (> 12 months), 20.6% (856/4164) of PWE discontinued PER due to AEs. Overall, 9.6% (414/4294) of PWE discontinued PER due to AEs and had psychiatric AEs (although it was not possible to determine in all PWE whether the withdrawal of PER was exclusively due to the psychiatric AEs). The most frequent psychiatric AEs (≥ 1% of PWE) in those who discontinued PER due to AEs were irritability (3.1%), behavioral disorders (2.8%) and mood disturbance (1.1%). No cases of homicidal ideation were reported.

**Table 3:** Summary of AEs (Tolerability Population)

Total PWE	*N* = 4617
PWE with any AE	
*N*^a^	4617
*n* (%)	2303 (49.9)
Most frequently reported AEs^b^	
*N*^a^	4617
Dizziness/vertigo, *n* (%)	701 (15.2)
Somnolence, *n* (%)	491 (10.6)
Irritability, *n* (%)	386 (8.4)
Behavioral disorders, *n* (%)	249 (5.4)
Instability/ataxia, *n* (%)	188 (4.1)
Fatigue, *n* (%)	170 (3.7)
Mood disturbance, *n* (%)	100 (2.2)
Weight increased, *n* (%)	94 (2.0)
Headache, *n* (%)	77 (1.7)
Anxiety, *n* (%)	70 (1.5)
Aggression, *n* (%)	64 (1.4)
Depression, *n* (%)	55 (1.2)
PWE with AEs leading to discontinuation	
12 months	
*N*^a^	4201
*n* (%)	739 (17.6)
Longer term (> 12 months)	
*N*^a^	4164
*n* (%)	856 (20.6)
PWE with any psychiatric AE	
*N*^a^	4590
*n* (%)	965 (21.0)
PWE with psychiatric AEs leading to discontinuation	
*N*^a^	4294
*n* (%)	414 (9.6)
Most frequently reported psychiatric AEs leading to discontinuation^b^	
*N*^a^	4617
Irritability, *n* (%)	141 (3.1)
Behavioral disorders, *n* (%)	127 (2.8)
Mood disturbance, *n* (%)	53 (1.1)

*AE* adverse event, *PWE* people with epilepsy

^a^Number of PWE for whom datum in question was available

^b^ > 1% of PWE

Results of bivariable analysis of the associations of baseline characteristics with occurrence of AEs are presented in Supplementary Table 3 (baseline characteristics selected for inclusion in the model to predict occurrence of AEs [i.e. those with *p* < 0.10] are indicated as shaded cells). It was not possible to obtain a multivariate model to predict baseline characteristics associated with the presence/absence of AEs.

## Discussion

The results of the PERMIT study demonstrate that PER was effective and generally well tolerated when used to treat focal or generalized epilepsy in everyday clinical practice. After 12 months of PER treatment, 64.2% of PWE were retained on PER, 58.3% had responded to treatment (≥ 50% reduction in seizure frequency from baseline), and 23.2% had been seizure free for at least 6 months. In addition, PER brought seizures under control in 52.7% of PWE with status epilepticus. There were significant reductions from baseline to the last visit in the monthly frequencies of total seizures, all types of focal and generalized seizures, and days with myoclonic seizures. A low proportion of PWE experienced seizure worsening after initiating PER treatment (6.6% at 12 months).

An important finding from the study was that PER was generally well tolerated when used over the long term in clinical practice. Approximately half the population experienced AEs at some point during follow-up, and the most frequently reported AEs (dizziness/vertigo, somnolence, irritability, behavioral disorders, instability/ataxia and fatigue) were consistent with those reported in clinical trials [5, 8–12]. The incidence of AEs was significantly higher in PWE for whom a fast PER titration was used (2 mg/week), compared with those for whom a slow titration was used (less than 2 mg/week), supporting evidence from previous clinical practice studies demonstrating lower incidences of AEs and AEs leading to discontinuation when PER was titrated more slowly than the rigid titration schedules employed in randomized clinical trials [58, 59]. Psychiatric AEs, which were reported as a common side effect in clinical trials (affecting ≥ 1/100 to < 1/10 PWE) [5], were experienced by 21.0% of PWE in PERMIT, and 9.6% of PWE who discontinued PER due to AEs had psychiatric AEs. Since a significant association was found between the incidence of psychiatric AEs and the presence of previous psychiatric comorbidity, the higher incidence of psychiatric AEs observed in PERMIT, in comparison with clinical trials, is likely to reflect the fact that clinical trials typically exclude PWE with psychiatric comorbidities [13, 15], whereas almost a quarter of PWE in PERMIT had psychiatric comorbidities at baseline (23.8%). Indeed, other evidence has indicated that PER-associated psychiatric and behavioral symptoms vary depending on the type of psychiatric and behavioral comorbidities present [60]. Although PER might aggravate some pre-existing psychiatric and behavioral symptoms, other such symptoms may improve with PER therapy [60]. It is noteworthy that there were no cases of homicidal ideation in PERMIT, some cases of which were reported in clinical trials [5, 61], illustrating that an individualized approach to dosage and titration can improve outcomes.

Evidence from clinical trials has shown that PER-associated AEs are more common during initial titration and appear to be dose-related; therefore, individuals should be monitored for these side effects, particularly during titration and at higher doses [62]. The overall incidence of AEs in PERMIT (49.9%) was lower than the rates reported in clinical trials, which ranged from 61.7% to 91.8% [8–12]. This is perhaps surprising, given that PWE treated in clinical practice are more diverse in terms of age and clinical characteristics, have higher levels of comorbidity and associated comedication, and may be more severe and refractory to treatment than those recruited for clinical trials [15]. However, this may in part be explained by the individualized approach to treatment used in clinical practice (in comparison with the defined dosing schedules employed in clinical trials), which is likely to result in improved tolerability. The proportion of PWE who discontinued due to AEs (17.6% after 12 months) was higher than most of the rates reported in clinical trials, which ranged from 2.9% to 19.0%; however, the durations of these trials were only 12–13 weeks [8–12]. PERMIT is the largest pooled analysis of PER clinical practice data conducted to date, with safety/tolerability assessed in over 4600 PWE; a far larger population than the total number of PWE included in PER clinical trials (approximately 1640) [5]. It is therefore reassuring that no new or unexpected safety signals emerged over the long term when PER was used under everyday clinical practice conditions.

Status epilepticus represents one of the most serious neurological emergencies [63] and encompasses a wide variety of subtypes and etiologies [64–67]. Benzodiazepines are typically used for first-line treatment of early status epilepticus, with intravenous ASMs administered as second-line therapy if seizures progress into established status epilepticus [66, 68–70]. ASMs commonly used in this setting include valproate, phenobarbital, phenytoin/fosphenytoin, levetiracetam and lacosamide, although there is no clear evidence for a preferred choice of second-line ASM therapy [66, 68, 69, 71]. Evidence for the use of PER in the treatment of status epilepticus is currently limited. In a systematic review (published in 2018) that included 10 articles and a total of 68 PWE, the rate of seizure control following treatment with PER ranged from 17 to 100% [72]. More recently, a single-center, retrospective, observational study of 75 PWE treated with PER for refractory status epilepticus reported a responder rate of 41.3% (response defined as clear resolution of the ictal pattern and/or seizures within 72 h of PER administration) [73]. In a further cohort study of 81 PWE treated with PER in intensive care for refractory or super-refractory status epilepticus, 33.3% responded to treatment [74]. In PERMIT, which included 74 PWE with status epilepticus from a wide range of clinical practice settings, the responder rate was 52.7%. PERMIT therefore provides valuable additional evidence indicating that PER may be a useful oral ASM therapy for some PWE with status epilepticus.

The large size of the PERMIT cohort allowed exploratory multivariable binary logistic regression analyses to be conducted, in order to try and identify baseline subject- and treatment-related factors that might help predict treatment outcomes. The baseline characteristics that were most associated with likelihood of retention were the use of a slow PER titration schedule and a relatively low number of previous ASMs, but the low specificity of the resulting model (10.7%) compromises its validity. Multivariable regression analyses of effectiveness revealed that the baseline factors most associated with response to PER treatment and seizure freedom were the same; these being the presence of a genetic etiology, lower number of concomitant ASMs at baseline, absence of psychiatric comorbidity, lower number of previous ASMs, and higher age of onset of epilepsy. The high association of effectiveness with a genetic etiology may have resulted from the cohort containing a relatively high proportion of individuals with IGE, since idiopathic epilepsies have a relatively benign disease course and/or show a favorable response to ASM therapy [75]: 12.4% of the PERMIT population had epilepsy with a genetic etiology and 12.6% had only generalized seizures at baseline, broadly consistent with previous reports that IGE represents 15–20% of all epilepsies [76]. PER, which is already approved for the treatment of primary generalized tonic–clonic seizures [5–7], was demonstrated to be an appropriate and effective treatment for these PWE, who were potentially relatively refractory (median number of previous ASMs 4.0) and with a less benign disease course than the wider IGE population. The observed association between effectiveness and a relatively low number of previous and concomitant ASMs is perhaps to be expected, since these characteristics are associated with PWE who are relatively early in their disease course and/or responsive to ASM therapy. The association between effectiveness and a higher age of onset of epilepsy may support previous findings indicating that ASMs have superior effectiveness in older versus younger people with newly diagnosed epilepsy [77–79]. Previous studies of other ASMs have demonstrated that the presence of psychiatric comorbidity in epilepsy may be associated with a poor response to ASM therapy [80–84] and it is therefore perhaps unsurprising that absence of psychiatric comorbidity was associated with greater PER effectiveness in the current study. Epilepsy and psychiatric disorders, including depression, may share common pathogenic mechanisms that result in cortical hyperexcitability, worsening response to pharmacotherapy; such mechanisms include increased glutamatergic activity [82, 85, 86]. The presence of psychiatric comorbidity may additionally impact the effectiveness of ASM therapy by increasing the likelihood of treatment non-adherence and/or by increasing stress, which is known to be a common trigger for seizures in individuals with epilepsy [82, 87, 88].

PERMIT additionally provided insights into how PER was dosed and titrated in clinical practice. The median dose of PER was 2.0 mg/day at treatment initiation and 6.0 mg/day at the last visit, in line with treatment guidelines, which recommend initiating PER at 2.0 mg/day and up-titrating to a maintenance dose of 4–8 mg/day (maximum recommended dose, 12 mg/day) in adults [5]. The median number of concomitant ASMs remained unchanged during the study (2.0 at baseline and last visit) and the proportion of PWE treated with PER as monotherapy was low (5.6% at baseline; 4.1% at the last visit). A fast titration (2 mg/week) was used in approximately two-thirds of PWE (65.5%) and a slow titration (less than 2 mg/week) was used in the remaining third (34.5%). As previously mentioned, fast titration was associated with a significantly higher incidence of AEs and, in multivariable analysis, halved the likelihood of retention, in comparison with slow titration. Retention is a composite effectiveness outcome reflecting both efficacy and safety/tolerability, which is particularly relevant to clinical practice [89, 90]. It is therefore pertinent to speculate that the outcomes observed in PERMIT may perhaps have been improved if a slow titration rate had more commonly been used.

The main limitation of this investigation was that it was a retrospective pooled analysis of studies that were heterogeneous in terms of their objectives and information reported. It therefore employed a statistical analysis approach that attempted to report information in the most complete and harmonized way possible; however, data were not available for all PWE at all timepoints, across all endpoints and assessments. In addition, the majority of studies included in PERMIT were retrospective analyses of cases, which were necessarily uncontrolled and likely to have selection bias against patients with greater likelihood of suffering known adverse effects, thus altering the balance between side effects and effectiveness, in comparison with blinded studies. It is also important to highlight that seizure freedom was defined as no seizures since at least the prior visit (rather than no seizures since initiation of PER treatment), which could have been 3 or 6 months, depending on the timepoint concerned. The relatively low retention rate over the longer term (29.5%), in comparison with the retention rate at 12 months (64.2%), is likely to reflect the fact that patients are typically followed up until a study drug is withdrawn, leading to an underestimation of long-term retention; therefore, the retention time (mean, 18.7 months) might be a more relevant outcome measure over the longer term than the retention rate. Although individual subject data were previously reviewed by the investigators of the original studies, they were not reviewed systematically post hoc in the current study. Multivariable binary logistic regression analyses were exploratory in nature and the models generated were limited in terms of both sensitivity and specificity; the findings of these analyses should be therefore be interpreted with caution, but nevertheless provide additional insights supporting the study’s other findings.

In summary, the PERMIT study demonstrated that PER is effective and generally well tolerated when used to treat people with focal and/or generalized epilepsy in everyday clinical practice. Including over 5000 PWE, PERMIT is the largest pooled analysis of PER clinical practice data conducted to date, and provides reassuring evidence of PER’s safety/tolerability, with no new or unexpected side effects emerging with long-term use in the real-world setting. Over 50% of PWE responded to PER therapy, including those with status epilepticus, and almost a quarter of PWE were seizure free for at least 6 months after 12 months of treatment. These findings complement evidence from clinical trials, further supporting the use of PER for the treatment of both focal and generalized epilepsy.

## Supplementary Information

Below is the link to the electronic supplementary material.

Supplementary file1 (DOCX 62 KB)

## Data Availability

The datasets generated and/or analyzed during the current study are available from the corresponding author on reasonable request.
