# Supplementary material for: PERMIT study: a global pooled analysis study of the effectiveness and tolerability of perampanel in routine clinical practice
Source: J Neurol. 2021 Aug 24;269(4):1957–77. doi: 10.1007/s00415-021-10751-y (PMC8940799; doi:10.1007/s00415-021-10751-y)
Supplement: Supplementary file 1 — Supplementary file1 (DOCX 62 KB) [file 415_2021_10751_MOESM1_ESM.docx]

**PERMIT study: A global pooled analysis study of the effectiveness and tolerability of perampanel in routine clinical practice**

**Vicente Villanueva^1^*, Wendyl D’Souza^2^, Hiroko Goji^3^, Dong Wook Kim^4^, Claudio Liguori^5^, Rob McMurray^6^, Imad Najm^7^, Estevo Santamarina^8^, Bernhard J. Steinhoff^9^, Pavel Vlasov^10^, Tony Wu^11^, Eugen Trinka^12–14^; on behalf of the PERMIT pooled analysis participants**

^1^Refractory Epilepsy Unit, Hospital Universitario y Politécnico La Fe, Valencia, Spain; ^2^Department of Medicine, St Vincent's Hospital Melbourne, The University of Melbourne, Victoria, Australia (ORCID: https://orcid.org/0000-0002-1750-5131); ^3^Neuropsychiatric Department, Aichi Medical University, Aichi, Japan; ^4^Department of Neurology, Konkuk University School of Medicine, Seoul, Korea; ^5^Epilepsy Centre, Neurology Unit, University Hospital “Tor Vergata”, Rome, Italy, and Department of Systems Medicine, University of Rome “Tor Vergata”, Rome, Italy (ORCID: https://orcid.org/0000-0003-2845-1332); ^6^European Knowledge Centre, Eisai Europe Ltd, Hatfield, Hertfordshire, UK; ^7^Cleveland Clinic Epilepsy Center, Neurological Institute, Cleveland Clinic, Cleveland, OH, USA; ^8^Epilepsy Unit, Hospital Universitari Vall d'Hebron, Barcelona, Spain (ORCID: https://orcid.org/0000-0003-1915-0335); ^9^Kork Epilepsy Center, Kehl-Kork, Germany, and Department of Neurology and Neurophysiology, Albert-Ludwigs University of Freiburg, Freiburg, Germany; ^10^Department of Neurology of General Medical Faculty of Moscow State University of Dentistry named after A.I. Evdokimov, Moscow, Russian Federation (ORCID: https://orcid.org/0000-0001-8321-5864); ^11^Chang Gung Memorial Hospital Linkou Medical Center and Chang Gung University College of Medicine, Taoyuan, Taiwan; ^12^Department of Neurology, Christian-Doppler University Hospital, Paracelsus Medical University, Centre for Cognitive Neuroscience, Salzburg, Austria; Affiliated EpiCARE Partner; ^13^Department of Public Health, Health Services Research and Health Technology Assessment, UMIT – University for Health Sciences, Medical Informatics and Technology, Hall in Tirol, Austria; ^14^Neuroscience Institute, Christian Doppler University Hospital, Paracelsus Medical University, Salzburg, Austria (ORCID: https://orcid.org/0000-0002-5950-2692)

***Corresponding author:**

Vicente Villanueva: villanueva_vichab@gva.es

**SUPPLEMENTARY MATERIAL**

**Supplementary Table 1. Bivariable analyses of relationships between baseline characteristics and retention (Retention Population).** Shaded cells indicate p-values <0.10, denoting baseline characteristics that were pre-selected for inclusion in multivariate logistic regression models

| **Characteristic** | **Retention** | **Discontinuation** | **p-value** |
| --- | --- | --- | --- |
| Sex  N^a^  Male, %  Female, % | 3215  51.4  48.6 | 1502  46.6  53.4 | 0.002 |
| Age, years  N^a^  Mean (SD)  Median (range) | 3102  39.3 (15.8)  38.0 (2.0–92.0) | 1453  40.2 (16.0)  39.0 (3.0–97.0) | 0.086 |
| Age category  N^a^  <12 years, %  12–<18 years, %  18–<65 years, %  ≥65 years, % | 3153  1.1  6.1  86.0  6.8 | 1453  1.0  5.1  86.2  7.8 | 0.373 |
| Age at epilepsy onset, years  N^a^  Mean (SD)  Median (range) | 2673  17.0 (17.6)  12.9 (0.0–92.0) | 1273  14.5 (16.7)  10.0 (0.0–97.0) | <0.001 |
| Duration of epilepsy, years  N^a^  Mean (SD)  Median (range) | 2731  22.7 (16.0)  20.4 (0.0–82.0) | 1320  25.3 (15.8)  23.0 (0.0–82.0) | <0.001 |
| Etiology (ILAE 2010 classification)  N^a^  Structural-metabolic, %  Unknown, %  Genetic, % | 2391  54.0  32.6  13.4 | 1174  60.1  30.7  9.1 | <0.001 |
| Etiology (ILAE 2017 classification)  N^a^  Structural, %  Genetic, %  Infectious, %  Unknown, %  Other (metabolic, immune, other), % | 2391  50.6  13.4  2.6  32.6  0.8 | 1174  56.0  9.1  3.2  30.7  1.0 | 0.001 |
| Epileptic syndrome  N^a^  No, %  Yes, % | 1643  80.0  20.0 | 676  86.5  13.5 | <0.001 |
| Vascular etiology  N^a^  No, %  Yes, % | 1643  95.3  4.7 | 676  96.7  3.3 | 0.121 |
| Tumor etiology  N^a^  No, %  Yes, % | 1643  94.3  5.7 | 676  95.1  4.9 | 0.419 |
| Learning disability  N^a^  No, %  Yes, % | 1617  71.9  28.1 | 854  71.2  28.8 | 0.726 |
| Psychiatric comorbidity  N^a^  No, %  Yes, % | 1695  77.8  22.2 | 799  71.5  28.5 | 0.001 |
| Seizure type  N^a^  Focal, %  Generalized, %  Both focal and generalized, %  Status epilepticus, % | 3074  81.2  14.3  3.8  0.7 | 1405  87.0  8.2  4.7  0.1 | <0.001 |
| Number of previous ASMs  N^a^  Mean (SD)  Median (range) | 2519  4.5 (3.8)  4.0 (0.0–19.0) | 1241  5.7 (4.0)  5.0 (0.0–19.0) | <0.001 |
| Number of concomitant ASMs  N^a^  Mean (SD)  Median (range) | 3081  2.2 (1.1)  2.0 (0.0–7.0) | 1480  2.4 (1.1)  2.0 (0.0–7.0) | <0.001 |
| Use of PER as early and late add-on therapy  N^a^  Late add-on, %  Early add-on, % | 1691  70.6  29.4 | 767  82.5  17.5 | <0.001 |
| Use of PER as monotherapy  N^a^  No, %  Yes, % | 3081  95.7  4.3 | 1480  97.6  2.4 | 0.001 |
| Use of levetiracetam  N^a^  No, %  Yes, % | 2977  62.7  37.3 | 1431  63.7  36.3 | 0.528 |
| Use of enzyme inducers  N^a^  No, %  Yes, % | 2977  50.0  50.0 | 1431  46.8  53.2 | 0.042 |
| Use of sodium channel blockers  N^a^  No, %  Yes, % | 2977  29.5  70.5 | 1431  24.5  75.5 | <0.001 |
| Use of ASMs targeting the GABA system  N^a^  No, %  Yes, % | 2977  69.4  30.6 | 1431  62.4  37.6 | <0.001 |
| Use of calcium channel blockers  N^a^  No, %  Yes, % | 2977  94.6  5.4 | 1431  94.0  6.0 | 0.416 |
| Use of potassium channel blockers  N^a^  No, %  Yes, % | 2977  97.9  2.1 | 1431  98.0  2.0 | 0.674 |
| Use of synaptic vesicle protein-2 modulators  N^a^  No, %  Yes, % | 2977  62.1  37.9 | 1431  63.4  36.6 | 0.402 |
| Use of ASMs with a mixed mode of action  N^a^  No, %  Yes, % | 2977  57.4  42.6 | 1431  56.0  44.0 | 0.380 |
| Use of slow and fast titration of PER  N^a^  Slow titration, %  Fast titration, % | 1185  42.0  58.0 | 641  22.2  77.8 | <0.001 |
| Final PER dose ≤4 mg/day  N^a^  Final PER dose ≤4 mg/day, %  Final PER dose >4 mg/day, % | 2616  32.1  67.9 | 528  57.0  43.0 | <0.001 |
| Final PER dose ≤6 mg/day  N^a^  Final PER dose ≤6 mg/day, %  Final PER dose >6 mg/day, % | 2616  58.8  41.2 | 528  77.5  22.5 | <0.001 |

^a^Number of PWE for whom datum in question was available.

AE, adverse event; ASM, antiseizure medication; GABA, gamma-aminobutyric acid; ILAE, International League Against Epilepsy; PER, perampanel; PWE, people with epilepsy; SD, standard deviation.

**Supplementary Table 2. Bivariable analyses of relationships between baseline characteristics and effectiveness: (A) Response to PER treatment (Effectiveness Population) and (B) Seizure freedom (Effectiveness Population).** Shaded cells indicate p-values <0.10, denoting baseline characteristics that were pre-selected for inclusion in multivariate logistic regression models. Seizure freedom was defined as no seizures since at least the prior visit. Response was defined as ≥50% seizure frequency reduction from baseline

| **(A) Response** |  |  |  |
| --- | --- | --- | --- |
| **Characteristic** | **Responder** | **Non-responder** | **p-value** |
| Sex  N^a^  Male, %  Female, % | 1928  51.8  48.2 | 1936  48.7  51.3 | 0.058 |
| Age, years  N^a^  Mean (SD)  Median (range) | 1897  39.6 (17.2)  38.0 (2.0–91.0) | 1902  37.8 (14.7)  37.0 (3.0–86.0) | <0.001 |
| Age category  N^a^  <12 years, %  12–<18 years, %  18–<65 years, %  ≥65 years, % | 1897  1.4  7.2  81.9  9.4 | 1902  1.2  6.3  88.5  4.0 | <0.001 |
| Age at epilepsy onset, years  N^a^  Mean (SD)  Median (range) | 1527  19.9 (19.2)  15.0 (0.0–90.0) | 1689  12.1 (14.6)  8.0 (0.0–84.9) | <0.001 |
| Duration of epilepsy, years  N^a^  Mean (SD)  Median (range) | 1566  20.2 (15.9)  16.0 (0.0–82.0) | 1724  25.8 (15.8)  24.0 (0.0–82.0) | <0.001 |
| Etiology (ILAE 2010 classification)  N^a^  Structural-metabolic, %  Unknown, %  Genetic, % | 1401  48.3  30.7  21.0 | 1501  60.6  32.2  7.1 | <0.001 |
| Etiology (ILAE 2017 classification)  N^a^  Structural, %  Genetic, %  Infectious, %  Unknown, %  Other (metabolic, immune, other), % | 1401  45.6  21.0  2.3  30.7  0.4 | 1501  55.6  7.1  3.7  32.2  1.3 | <0.001 |
| Epileptic syndrome  N^a^  No, %  Yes, % | 1077  73.6  26.4 | 890  88.2  11.8 | <0.001 |
| Vascular etiology  N^a^  No, %  Yes, % | 1077  94.2  5.8 | 890  97.1  2.9 | 0.002 |
| Tumor etiology  N^a^  No, %  Yes, % | 1077  92.7  7.3 | 890  95.6  4.4 | 0.006 |
| Learning disability  N^a^  No, %  Yes, % | 790  73.5  26.5 | 965  63.8  36.2 | <0.001 |
| Psychiatric comorbidity  N^a^  No, %  Yes, % | 1057  79.2  20.8 | 800  72.4  27.6 | 0.001 |
| Seizure type  N^a^  Focal, %  Generalized, %  Both focal and generalized, % | 1821  76.8  19.4  3.7 | 1805  88.3  7.8  3.9 | <0.001 |
| Number of previous ASMs  N^a^  Mean (SD)  Median (range) | 1424  3.6 (3.4)  3.0 (0.0–18.0) | 1546  5.8 (4.0)  5.0 (0.0–19.0) | <0.001 |
| Number of concomitant ASMs  N^a^  Mean (SD)  Median (range) | 1865  1.9 (1.1)  2.0 (0.0–6.0) | 1811  2.5 (1.0)  2.0 (0.0–0.7) | <0.001 |
| Use of PER as early and late add-on therapy  N^a^  Late add-on, %  Early add-on, % | 934  54.3  45.7 | 1044  84.9  15.1 | <0.001 |
| Use of PER as monotherapy  N^a^  No, %  Yes, % | 1865  91.6  8.4 | 1811  98.7  1.3 | <0.001 |
| Use of levetiracetam  N^a^  No, %  Yes, % | 1818  65.1  34.9 | 1734  63.4  36.6 | 0.293 |
| Use of enzyme inducers  N^a^  No, %  Yes, % | 1818  59.1  40.9 | 1734  39.7  60.3 | <0.001 |
| Use of sodium channel blockers  N^a^  No, %  Yes, % | 1818  39.8  60.2 | 1734  17.9  82.1 | <0.001 |
| Use of ASMs targeting the GABA system  N^a^  No, %  Yes, % | 1818  76.2  23.8 | 1734  60.2  39.8 | <0.001 |
| Use of calcium channel blockers  N^a^  No, %  Yes, % | 1818  96.3  3.7 | 1734  94.2  5.8 | 0.004 |
| Use of potassium channel blockers  N^a^  No, %  Yes, % | 1818  99.1  0.9 | 1734  96.6  3.4 | <0.001 |
| Use of synaptic vesicle protein-2 modulators  N^a^  No, %  Yes, % | 1818  64.4  35.6 | 1734  62.3  37.7 | 0.200 |
| Use of ASMs with a mixed mode of action  N^a^  No, %  Yes, % | 1818  59.8  40.2 | 1734  58.1  41.9 | 0.315 |
| Use of slow and fast titration of PER  N^a^  Slow titration, %  Fast titration, % | 747  46.3  53.7 | 924  26.4  73.6 | <0.001 |
| Final PER dose ≤4 mg/day  N^a^  Final PER dose ≤4 mg/day, %  Final PER dose >4 mg/day, % | 1664  37.8  62.2 | 1310  26.7  73.3 | <0.001 |
| Final PER dose ≤6 mg/day  N^a^  Final PER dose ≤6 mg/day, %  Final PER dose >6 mg/day, % | 1664  66.0  34.0 | 1310  52.0  48.0 | <0.001 |
| **(B) Seizure freedom** |  |  |  |
| **Characteristic** | **Seizure free** | **Not seizure free** | **p-value** |
| Sex  N^a^  Male, %  Female, % | 867  50.1  49.9 | 3359  50.0  50.0 | 0.982 |
| Age, years  N^a^  Mean (SD)  Median (range) | 865  40.7 (18.2)  39.0 (2.0–91.0) | 3309  38.6 (15.1)  38.0 (3.0–90.0) | 0.002 |
| Age category  N^a^  <12 years, %  12–<18 years, %  18–<65 years, %  ≥65 years, % | 865  1.4  6.6  80.2  11.8 | 3309  1.1  6.0  87.7  5.1 | <0.001 |
| Age at epilepsy onset, years  N^a^  Mean (SD)  Median (range) | 698  24.1 (20.0)  18.0 (0.0–90.0) | 2777  13.4 (15.3)  9.0 (0.0–85.0) | <0.001 |
| Duration of epilepsy, years  N^a^  Mean (SD)  Median (range) | 704  16.7 (15.4)  12.0 (0.0–82.0) | 2831  25.2 (15.7)  23.0 (0.0–82.0) | <0.001 |
| Etiology (ILAE 2010 classification)  N^a^  Structural-metabolic, %  Unknown, %  Genetic, % | 628  43.3  23.1  33.6 | 2528  58.0  34.1  7.9 | <0.001 |
| Etiology (ILAE 2017 classification)  N^a^  Structural, %  Genetic, %  Infectious, %  Unknown, %  Other (metabolic, immune, other), % | 628  41.6  33.6  1.6  23.1  0.2 | 2528  54.0  7.9  3.1  34.1  0.9 | <0.001 |
| Epileptic syndrome  N^a^  No, %  Yes, % | 536  63.1  36.9 | 1558  87.0  13.0 | <0.001 |
| Vascular etiology  N^a^  No, %  Yes, % | 536  91.6  8.4 | 1558  96.8  3.2 | <0.001 |
| Tumor etiology  N^a^  No, %  Yes, % | 536  92.4  7.6 | 1558  94.9  5.1 | 0.027 |
| Learning disability  N^a^  No, %  Yes, % | 313  87.5  12.5 | 1748  67.1  32.9 | <0.001 |
| Psychiatric comorbidity  N^a^  No, %  Yes, % | 591  82.1  17.9 | 1353  74.6  25.4 | <0.001 |
| Seizure type  N^a^  Focal, %  Generalized, %  Both focal and generalized, % | 840  65.2  31.1  3.7 | 3123  88.3  8.3  3.3 | <0.001 |
| Number of previous ASMs  N^a^  Mean (SD)  Median (range) | 594  2.4 (2.5)  2.0 (0.0–16.0) | 2663  5.5 (3.9)  5.0 (0.0–19.0) | <0.001 |
| Number of concomitant ASMs  N^a^  Mean (SD)  Median (range) | 844  1.5 (1.0)  1.0 (0.0–6.0) | 3190  2.4 (1.0)  2.0 (0.0–7.0) | <0.001 |
| Use of PER as early and late add-on therapy  N^a^  Late add-on, %  Early add-on, % | 373  36.7  63.3 | 1784  80.2  19.8 | <0.001 |
| Use of PER as monotherapy  N^a^  No, %  Yes, % | 844  86.0  14.0 | 3190  98.0  2.0 | <0.001 |
| Use of levetiracetam  N^a^  No, %  Yes, % | 826  62.1  37.9 | 3051  64.5  35.5 | 0.197 |
| Use of enzyme inducers  N^a^  No, %  Yes, % | 826  70.5  29.5 | 3051  43.0  57.0 | <0.001 |
| Use of sodium channel blockers  N^a^  No, %  Yes, % | 826  54.0  46.0 | 3051  21.3  78.7 | <0.001 |
| Use of ASMs targeting the GABA system  N^a^  No, %  Yes, % | 826  84.3  15.7 | 3051  63.2  36.8 | <0.001 |
| Use of calcium channel blockers  N^a^  No, %  Yes, % | 826  97.6  2.4 | 3051  94.1  5.9 | <0.001 |
| Use of potassium channel blockers  N^a^  No, %  Yes, % | 826  99.8  0.2 | 3051  97.2  2.8 | <0.001 |
| Use of synaptic vesicle protein-2 modulators  N^a^  No, %  Yes, % | 826  61.7  38.3 | 3051  63.7  36.3 | 0.313 |
| Use of ASMs with a mixed mode of action  N^a^  No, %  Yes, % | 826  64.5  35.5 | 3051  56.8  43.2 | <0.001 |
| Use of slow and fast titration of PER  N^a^  Slow titration, %  Fast titration, % | 357  50.7  49.3 | 1326  33.7  66.3 | <0.001 |
| Final PER dose ≤4 mg/day  N^a^  Final PER dose ≤4 mg/day, %  Final PER dose >4 mg/day, % | 779  51.9  48.1 | 2297  27.3  72.7 | <0.001 |
| Final PER dose ≤6 mg/day  N^a^  Final PER dose ≤6 mg/day, %  Final PER dose >6 mg/day, % | 779  79.2  20.8 | 2297  54.1  45.9 | <0.001 |

^a^Number of PWE for whom datum in question was available.

AE, adverse event; ASM, antiseizure medication; GABA, gamma-aminobutyric acid; ILAE, International League Against Epilepsy; PER, perampanel; PWE, people with epilepsy; SD, standard deviation.

**Supplementary Table 3. Bivariable analyses of relationships between baseline characteristics and tolerability (Tolerability Population).** Shaded cells indicate p-values <0.10, denoting baseline characteristics that were pre-selected for inclusion in multivariate logistic regression models

| **Characteristic** | **No AEs** | **AEs** | **p-value** |
| --- | --- | --- | --- |
| Sex  N^a^  Male, %  Female, % | 2306  52.0  48.0 | 2296  45.9  54.1 | <0.001 |
| Age, years  N^a^  Mean (SD)  Median (range) | 2232  39.0 (16.4)  38.0 (2.0–92.0) | 2211  40.3 (15.7)  39.0 (4.0–97.0) | 0.010 |
| Age category  N^a^  <12 years, %  12–<18 years, %  18–<65 years, %  ≥65 years, % | 2270  1.6  6.5  84.3  7.6 | 2233  0.9  4.9  86.9  7.3 | 0.012 |
| Age at epilepsy onset, years  N^a^  Mean (SD)  Median (range) | 1719  16.3 (17.3)  12.0 (0.0–92.0) | 1923  15.4 (17.0)  11.0 (0.0–97.0) | 0.018 |
| Duration of epilepsy, years  N^a^  Mean (SD)  Median (range) | 1759  22.5 (15.9)  20.0 (0.0–77.0) | 1989  24.8 (16.2)  23.0 (0.0–82.0) | <0.001 |
| Etiology (ILAE 2010 classification)  N^a^  Structural-metabolic, %  Unknown, %  Genetic, % | 1512  54.4  30.0  15.6 | 1799  56.8  32.4  10.8 | <0.001 |
| Etiology (ILAE 2017 classification)  N^a^  Structural, %  Genetic, %  Infectious, %  Unknown, %  Other (metabolic, immune, other), % | 1512  50.0  15.6  3.4  30.0  1.0 | 1799  53.2  10.8  2.8  32.4  0.8 | 0.001 |
| Epileptic syndrome  N^a^  No, %  Yes, % | 1146  80.1  19.9 | 1110  80.6  19.4 | 0.753 |
| Vascular etiology  N^a^  No, %  Yes, % | 1146  93.5  6.5 | 1110  95.9  4.1 | 0.011 |
| Tumor etiology  N^a^  No, %  Yes, % | 1146  92.8  7.2 | 1110  96.2  3.8 | <0.001 |
| Learning disability  N^a^  No, %  Yes, % | 1030  69.0  31.0 | 1194  71.8  28.2 | 0.157 |
| Psychiatric comorbidity  N^a^  No, %  Yes, % | 1146  81.8  18.2 | 1186  70.0  30.0 | <0.001 |
| Seizure type  N^a^  Focal, %  Generalized, %  Both focal and generalized, %  Status epilepticus, % | 2163  79.8  14.9  2.1  3.2 | 2141  83.8  12.0  3.9  0.2 | <0.001 |
| Number of previous ASMs  N^a^  Mean (SD)  Median (range) | 1681  4.3 (3.7)  3.0 (0.0–19.0) | 1887  5.2 (3.9)  5.0 (0.0–19.0) | <0.001 |
| Number of concomitant ASMs  N^a^  Mean (SD)  Median (range) | 2132  2.2 (1.2)  2.0 (0.0–7.0) | 2212  2.3 (1.1)  2.0 (0.0–7.0) | 0.002 |
| Use of PER as early and late add-on therapy  N^a^  Late add-on, %  Early add-on, % | 1195  69.6  30.4 | 1191  78.7  21.3 | <0.001 |
| Use of PER as monotherapy  N^a^  No, %  Yes, % | 2132  94.9  5.1 | 2212  96.0  4.0 | 0.085 |
| Use of levetiracetam  N^a^  No, %  Yes, % | 2037  62.2  37.8 | 2147  63.1  36.9 | 0.564 |
| Use of enzyme inducers  N^a^  No, %  Yes, % | 2037  49.0  51.0 | 2147  49.0  51.0 | 0.997 |
| Use of sodium channel blockers  N^a^  No, %  Yes, % | 2037  30.2  69.8 | 2147  26.9  73.1 | 0.016 |
| Use of ASMs targeting the GABA  system  N^a^  No, %  Yes, % | 2037  70.0  30.0 | 2147  64.4  35.6 | <0.001 |
| Use of calcium channel blockers  N^a^  No, %  Yes, % | 2037  95.4  4.6 | 2147  93.8  6.2 | 0.020 |
| Use of potassium channel blockers  N^a^  No, %  Yes, % | 2037  98.3  1.7 | 2147  97.8  2.2 | 0.186 |
| Use of synaptic vesicle protein-2 modulators  N^a^  No, %  Yes, % | 2037  61.3  38.7 | 2147  62.6  37.4 | 0.375 |
| Use of ASMs with a mixed mode of action  N^a^  No, %  Yes, % | 2037  57.4  42.6 | 2147  56.4  43.6 | 0.521 |
| Use of slow and fast titration of PER  N^a^  Slow titration, %  Fast titration, % | 738  46.2  53.8 | 941  31.3  68.7 | <0.001 |
| Final PER dose ≤4 mg/day  N^a^  Final PER dose ≤4 mg/day, %  Final PER dose >4 mg/day, % | 1795  32.1  67.9 | 1343  40.6  59.4 | <0.001 |
| Final PER dose ≤6 mg/day  N^a^  Final PER dose ≤6 mg/day, %  Final PER dose >6 mg/day, % | 1795  57.3  42.7 | 1343  67.4  32.6 | <0.001 |

^a^Number of PWE for whom datum in question was available.

AE, adverse event; ASM, antiseizure medication; GABA, gamma-aminobutyric acid; ILAE, International League Against Epilepsy; PER, perampanel; PWE, people with epilepsy; SD, standard deviation.
